# Brain Oxygenation in Post-concussion Combat Sport Athletes

**DOI:** 10.3389/fspor.2021.725096

**Published:** 2021-11-30

**Authors:** Paolo Tiberini, Giuseppe D'Antona, Antonio Cicchella

**Affiliations:** ^1^Department of Movement Sciences and Well-being, University of Naples Parthenope, Naples, Italy; ^2^Criams-Sport Medicine Centre Voghera, University of Pavia, Pavia, Italy; ^3^Department of Public Health, Experimental and Forensic Medicine, University of Pavia, Pavia, Italy; ^4^Department for Quality of Life Studies, University of Bologna, Bologna, Italy

**Keywords:** brain concussion, mild traumatic brain injuries (mTBI), sway analysis, combat sport athletes, boxing athletes, brain concussion [MeSH], medio-lateral oscillation, near infrared spectroscopy (NIRS)

## Abstract

**Purpose:** Investigate the feasibility of a non-invasive method to evaluate the physical and cognitive repercussions of long-lasting post-concussion effects in professional combat sports athletes. To help athletes return to professional combat, there is a need for unbiased objective tools and techniques used as a prognostic method of recovery after Sport Related Concussion (SRC).

**Methods:** Six mild Traumatic Brain Injury (mTBI) athletes, age 20 ÷ 43 yr (1 female, 5 males) and 7 not concussed (NC) participants (amateur), age 24 ÷ 38 yr (3 females, 4 males), were tested Inspired/expired gas concentration, Cerebral changes in oxygenated hemoglobin (Δ[HbO_2_]) and deoxygenated hemoglobin (Δ[HHb]) were measured using near infrared spectroscopy (NIRS) with a 3-step protocol: rest before maximal oxygen uptake (VO_2_max) test, hypercapnia, and recovery after VO_2_max test. The brain oxygenation and respiratory parameters of both sample sets were calculated using a non-parametric test (Mann-Whitney U test). Aerobic fitness outcome was quantified through mean average using the Bruce test. Participants performed Fitt's test using a laptop and analysis of medio-lateral and anterior-posterior range of oscillation was carried out via a force platform Romberg test.

**Results:** mTBI group showed statistically significant differences in saturated hemoglobin Δ[HbO_2_] (*p* < 0.001) during rest and recovery phase after maximal incremental exercise, in medio-lateral sway eyes open (*p* = 0.008, NC 25.35 ± 4.11 mm and mTBI 17.65 ± 4.79 mm). VO_2_max revealed no significant differences between the two groups: NC 47.47 ± 4.91 mTBI 49.58 ± 5.19 ml/kg/min^−1^. The 2 groups didn't differ for maximum power output (NC 220 ± 34, mTBI 255 ± 50 W). End-tidal fractional concentration of O_2_ (FetO_2_ NC15.20 ± 0.41, mTBI 16.09 ± 0.68) throughout hypercapnia, saturated blood hemoglobin (Δ[HbO_2_]) revealed significant differences with the mTBI group. No differences emerged from Fitt's test.

**Conclusions:** It emerges that NIRS is able to reveal differences in long time outcomes of mTBI. The medio-lateral variations cannot be considered as a marker of long-term damage in athletes specifically trained for balance.

## Introduction

Traumatic Brain Injuries (TBIs) can be distinguished based on the severity in: mild, moderate, and severe (Kaj, 2016). A concussion is considered as part of the mild TBI spectrum associated diseases and often the term is used as synonymous of mTBI. Generally, the term Sport-Related Concussion” (SRC) is used to represent the immediate and transient symptoms of TBI. mTBIs are the less severe brain trauma and, generally, spontaneously resolve after a short period of recovery (McCrory et al., 2017) and represent 70–90% of all treated brain injuries (Cassidy, 2004). The sequelae of chronic subclinical neurological effects of recurrent concussions are not well-understood. In the long term, it has been observed that repetitive cerebral concussions could be a possible cause of dementia-related syndromes onset (Guskiewicz Kevin, 2005) and other coexistent diseases. Concussions can also lead to psychiatric and psychological disorders (Finkbeiner et al., 2016). The effects on physical performance, on motor learning and on the coordination of movements in sub-clinical stages of post-concussion events are partly known but not yet clarified. Some studies show that there are alterations of oculomotor functions in those subjects who have suffered traumas during sports (Hecimovich et al., 2019), as a consequence of the cognitive performance decline, a slowdown in physical activity and reflexes has been observed as for example an impaired speed of movement during the walk test (Howell et al., 2019). Also, the execution of psychomotor tasks may slow in multiple-concussed athletes compared to the non-concussed. In the long run (Beaulieu et al., 2019), in addition to migraine, other concomitant conditions, such as anxiety and vestibular problems below the threshold of formal medical diagnosis-symptoms (Kontos et al., 2019), may be found. A lower incidence rate of head injuries has been also observed in more aerobically trained subjects than in less trained, even if this difference does not change the effects of neuro-cognitive decline given by a possible concussion (Kontos et al., 2006). Measurements performed with transcranial ultrasonographic doppler, showed the changes in cerebral circulation occurring in concussed subjects (Len et al., 2011). During daily life, comparable to rest, there are no persistent changes. Instead, during tests in conditions of hypo- and hypercapnia, the concussed subjects showed a fall in cerebral diffusion speed as well as an increase in recovery times compared to healthy subjects (Len et al., 2011). The limited perfusion of oxygen, determined by the autonomic nervous system, can cause limitations of brain functions or high metabolic demands to an already partially compromised brain to guarantee the required oxygen request (Jünger et al., 1997).

### Post-concussive Symptoms

After a closed head injury, during which the skull and dura mater remain intact, the aforementioned symptoms may lead to post-concussion syndrome, a mild form of TBI (MeSH subject heading scope note, 2003) that could be chronic, permanent, or late-emerging. Even, some low-grade effects of concussion, such as impairments of balance, precision, attention, working memory, and oxygenation can be observed (Brush et al., 2018).

The mTBI is a complex injury that caused a large number of subjects, the development of persistent symptoms so far (Brush et al., 2018).

### Persistent Post-concussive Symptoms

The “punch-drunk syndrome” also called *dementia pugilistica*, was first described in 1928 (Martland, 1928). Since then, in 2009 the VA/DoD (Veterans Administration/Department of Defense) draw up a clinical practice guideline underlining that “there is no consensus on a case definition for persistent symptoms attributed to concussion/mTBI and no consensus on the time course when acute symptoms should be considered persistent” (Cifu et al., 2009). To date, no definitive studies demonstrated the incidence of persistent symptoms after repeated concussions and no common agreement on the development of persistent symptoms.

(Guskiewicz Kevin, 2005), observed that retired professional football player that suffered three or more reported concussion had a 5-fold prevalence of problems during a questionnaire focusing on memory and issues related to Mild Cognitive Impairment (MCI) compared with retirees without a history of concussion. They also found an association between subjects who suffered a concurrent concussion during their life and an earlier onset of Alzheimer's disease. Even on the adolescent, mTBI produced cognitive function reduction, in particular verbal memory, visual memory and impulse control (Taylor et al., 2018). The cognitive functions in adolescents are compromised in performance caused by persistent effects than previously thought. Moreover, tested on a population of 5,656 adolescent aged 13–19 years, over a period of 5 years, the age at which an individual has his or her first concussion should be a related factor that influences the long-lasting effects of concussion (Taylor et al., 2018).

There is a large number of subjects such as youth and high school students or professional athletes participating in contact sports that every year are involved in repetitive brain trauma; CTE represents an important public health issue that should be considered as diseases acknowledged by the public institutions (Stern et al., 2011). A study of Neary et al. (2020) showed how NIRS (Near-Infrared Spectroscopy) can be used as a diagnostic tool in the acute assessment of concussion (1 week post-injury) but no study exists in the long term assessment of the outcomes of mTBI. The article lays the groundwork for the present research as its shows that objective measurements of cerebral hemodynamics can be performed by NIRS.

### Aim of Research

There is a lack of studies monitoring the long-term cognitive impairment (e.g., anxiety, loss of concentration) after recurrent concussions and their neuropsychological consequences and/or behavioral impairments in several sport categories as boxing, ski, snowboarding, rugby, or American football.

The long-term neuromotor functions in concussed subjects have been poorly investigated in the literature.

The aims of the present study were to test a non-invasive method to evaluate the physical and cognitive behaviors following long-lasting concussion exposure in professional boxers and to investigate if there are differences between physiological (variation in Δ[HbO_2_]) and performance (VO_2_max) variables and see whether differences in these variables can be highlighted between concussed (mTBI) and not concussed (NC) participants. In particular, we assessed if concussed participants showed differences between brain oxygenation, balance, maximal aerobic capacity, and power, in comparison to non-concussed healthy participants. This method was adopted in part by Len et al. (2011) but only with regard to changes in Δ[HbO_2_] during rest and during hypercapnia; according to our knowledge, Δ[HHb] improvement has never been verified in the same test and never monitored during the recovery phase after maximal incremental physical exercise test in order to monitor the behavior of tissue oxygenation in mTBI participants.

### Hypothesis

Our hypothesis were: significant differences exist between NC practitioners and people with mTBI in the mid-lateral oscillation, Δ[HbO2] and Δ[HHb] during the three phases of the test (Rest, Hypercapnea, Recovery).

## Methods

Thirteen participants (20–43 year old, male and female) participated in the study. Participants were recruited by public announcement addressed to gyms and specialist centers and were divided into two groups: not concussed participants (NC, *n* = 7) age 24÷38 yr (3 females, 4 males), boxers or fighters (3 boxers and 4 fighters) and repeated mTBI participants (mTBI, *n* = 6) age 20÷43 yr (1 female, 5 males) boxers and fighters (4 boxers and 2 fighters), anthropometry is reported in Table 1.

**Table 1:** Anthropometric and aerobic power data with mean values ± standard deviation values.

	**Age**	**Body mass**	**Height**	**BMI**	**VO_**2**_max/Kg**	**HRmax**	**Pmax**
NC (mean ± SD)	30 ± 5	74.76 ± 12.38	173 ± 9	24.7 ± 2,4	40.1 ± 4.9	173 ± 8	220 ± 34
mTBI (mean ± SD)	29 ± 9	73.38 ± 15.62	176 ± 6	23.4 ± 4.0	45.4 ± 5.2	173 ± 17	255 ± 50

*N.C, not Concussed participants; mTBI, Concussed participants; BMI, Body Mass Index; VO_2_maxexpressed in (ml/min/Kg); HRmax, heart rate expressed in bpm; Pmax (W), maximum power reached during CPET test*.

Inclusion criteria for the mTBI were age 20÷45 years of age, have experienced two or more mTBIs within the past 10 years but not within 6 months. Exclusion criteria for both groups included: substance abuse, major psychological disorders, the use of beta-blockers or calcium antagonists. Recruited participants were retired or professional boxers or fighters coming from martial arts like muay thai and mixed martial arts (MMA). Control participants did not experience repeated mTBIs during their entire life. Before the testing session, informed written consent was obtained by each participant. Procedures were approved by the research ethics board of the University of Bologna. Participants were asked to refrain to eat food, drink coffee ingestion, or smoke in the previous 3 h before tests or drink alcohol in the previous 12 h.

### Procedure

On the day of the experiment, the participant was subjected to five subsequent tests for a total duration of ~60 min: Romberg's test, reactivity and fine motor skills assessment, brain oxygenation measurement (Δ[HbO_2_]; Δ[HHb]), measurement of VO_2_max, cerebral oxygenation (Cox) during recovery.

First, Romberg's test required the participants to be barefoot [30 s with eyes open (eo) and 30 s with eyes closed (EC)] on a kistler platform mod. 9286B (Kistler, Winterthur, Switzerland). For balance assessment the Sway ver. 1.4 (BTS Bioengineering, Garbagnate Milanese, Italy) software was used and the medio-lateral oscillation (MLroo) and anterior posterior range of oscillations (AProo) were calculated (Prieto et al., 1996; Mirow et al., 2016) to evaluate the origin, ocular or vestibular, of possible alterations of the balance (Błaszczyk, 2016). For an evaluation of reactivity and fine motor skills, Fitt's standardized test (Fitts, 1954) was administered on a personal computer through the use of the software PEBL (Mueller and Piper, 2014) based on Fitt's law (Fitts, 1954). This “aimed movement test” (Mueller and Piper, 2014) was performed using the computer mouse to point targets of different sizes and distances that appeared on the computer screen. For each participant, we collected 210 trials and we used the time of 100 trials for comparison. The first 50 trials were considered as training, the trials 51–150 were retained, and the last 60 trials (from 151 to 210) were not considered. Every participant was not aware of which trials were taken for the analysis. Average reaction times were compared.

Brain oxygenation measurement was performed using a standardized protocol (Len et al., 2011; Bishop and Neary, 2018). In the first phase, the participant was asked to lay supine with eyes open in resting breathing condition, in order to measure the change in concentration of oxyhaemoglobin (Δ[HbO_2_])parameter used to suggest cerebral blood flow (CBF), deoxyhaemoglobin (Δ[HHb]) a reliable estimator of changes in tissue de-oxygenation status (Hoshi et al., 2001). VO_2_, VCO_2_, end-tidal fractional concentration of CO_2_ (FetCO_2_), end-tidal fractional concentration of O_2_ (FetO_2_) were also used.

In the second phase, a hypercapnia test was performed including 40 s of normal breathing and 20 s of breath retention for a total of 5 steps (5 min total), to allow to measure drift from the basal level (Bishop and Neary, 2018). The transition time from standing to lying between each phase has also been set to 60 s for each subject, lying on a massage table.

The measurements of oxyhaemoglobin (Δ[HbO_2_]) and deoxyhaemoglobin (Δ[HHb]) concentrations were done using an 85 × 20 mm transcranial probe, applied 2 cm above the root of the nose and connected with a near-infrared spectroscope (NIRS, Nirox S.r.l) just above the supraorbital ridge (Kleinschmidt et al., 1996). Based on the transparency principle of human tissue to the near-infrared radiation, it uses light in the spectrum from 650 to 1,000 nm. We used a probe equipped with three low-power laser diodes (<10 mW) that emit radiation at 685, 830, and 980 nm (interoptode distance 44 mm, accuracy ± 5 μM). The 40 Hz data sampling frequency was then filtered in order to reduce the amount of data and obtain two values per second according to Wolf et al. (2007).

The breath-by-breath gases were measured using a metabolimeter Quark CPET (Cosmed S.r.l, Bologna).

The aerobic power (VO_2_max) was calculated by means of a cardiopulmonary exercise Bruce ramp test (CPET) using a cycle ergometer (5 min of warming up at 80 W and then with an increase of 20 W every 2 min, until exhaustion). During CPET, the heart rate was continuously monitored with an electrocardiogram interfaced with the metabolic cart. The parameter measured were: VO_2_/Kg (ml/Kg/min), hearth rate HR(bpm), maximum power P(W).

During the recovery phase following the CPET test transcranial Δ[HbO_2_]/Δ[HHb] and respiratory gases were measured in supine posture.

### Statistical Analysis

The data were analyzed using the SPSS statistical software (ver. 23.0). Mean and SD values were calculated for Δ[HbO_2_], Δ[HHb] and FetO_2_, FetCO_2_ for each participant. A Kolmogorov-Smirnov test was performed to assess the normality of data, and a Mann-Whitney U test was performed to evaluate the differences between groups (mTBI vs. NC). Significance was set at *p* < 0.05.

## Results

**Figure 1:**
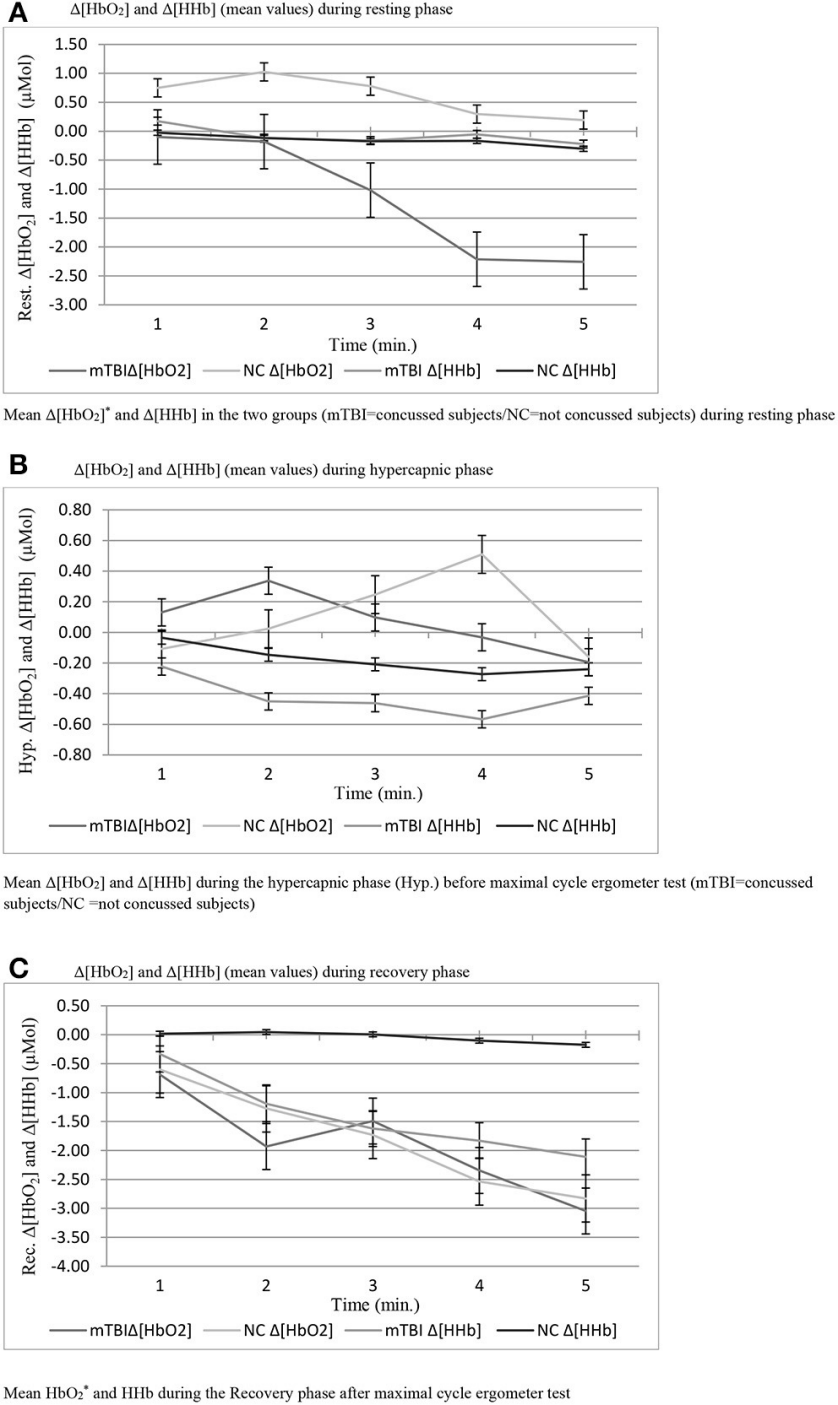
**(A)** Δ[HbO2] and Δ[HHb] (mean values) during resting phase. **(B)** Δ[HbO2] and Δ[HHb] (mean values) during hypercapnic phase. **(C)** Δ[HbO2] and Δ[HHb] (mean values) during recovery phase.

Anthropometric data and aerobic power are summarized in Table 1. No significant difference was found in VO_2_max/kg between groups (mTBI: 45.4 ± 5.2; NC: 40.1 ± 4.9 ml/kg/min^−1^). Sway test (Table 2) revealed a significant difference in medio-lateral sway at eyes open (*p* = 0.008) between mTBI (17.65 ± 4.79 mm) and NC (25.35 ± 4.11 mm). Mann-Whitney *U*-test showed a significant difference in FetO_2_ average during the hypercapnic phase (*p* = 0.008) (Table 3). During rest (Figure 1A) and during the recovery phase (Figure 1C) from CPET, NIRS showed a statistically significant difference in Δ[HbO_2_] (*p* < 0.001) between groups.

**Table 2:** Sway test.

	**MLroo(eo)**	**MLroo(ec)**	**AProo(eo)**	**AProo(ec)**	**TL(eo)**	**TL(ec)**
NC	25.3 ± 4.1	29.2 ± 9.8	22.1 ± 3.1	34.2 ± 10.5	485.5 ± 161.2	808.2 ± 236.9
mTBI	17.6 ± 4.8^*^	29.4 ± 10.2	18.7 ± 5.7	29.6 ± 7.7	381.6 ± 116.2	601.3 ± 256.1

*MLroo, Medio-Lateral range of oscillation; AProo, Anterior-Posterior range of oscillation; TL, Trace Length, eyes open (eo) and eyes closed (ec). All the measure are expressed in millimeter (mm)*.

* *statistically significant result*.

**Table 3:** FetO_2_ (fractional end tidal O_2_) and FetCO_2_ (fractional end tidal CO_2_) mean data during the three phases.

	**Rest phase**	**Hyp. phase**	**Rec. phase**
FetO_2_ NC	13.80 ± 3.90	15.20^*^± 0.41	16.37 ± 0.51
FetO_2_ mTBI	15.45 ± 1.32	16.09^*^± 0.68	16.44 ± 0.50
FetCO_2_ NC	6.51 ± 3.82	5.04 ± 0.37	4.48 ± 0.47
FetCO_2_ mTBI	5.14 ± 0.64	4.89 ± 0.59	4.53 ± 0.42

*FetO_2_ NC, Fractional End Tidal O_2_ Not Concussed participants; FetO_2_ mTBI, Fractional End Tidal O_2_ Concussed Subjects; FetCO_2_ NC, Fractional End Tidal CO_2_ Not Concussed participants; FetCO_2_ mTBI, Fractional End Tidal CO_2_ Concussed Subjects Rest phase (Rest Phase); Hypercapnic Phase (Hyp. phase), Recovery Phase (Rec. phase). Statistically significant between groups (p = 0.0008)*.

* *statistically significant result*.

No statistically significant differences were observed during the hypercapnic test regarding Δ[HbO_2_], Δ[HHb] (Figure 1B) and in reaction time that take into account total time and maximum trial duration in the Fitt's test (Table 4).

**Table 4:** Fitt's test data.

	**T max (ms)**	**Rt (ms)**	**Tt (s)**
NC	2,313 ± 460	1,003 ± 104	100.4 ± 10.4
mTBI	2,347 ± 621	1,060 ± 75	106.0 ± 7.5

*T max, Maximum Trial duration (mean of maximum ± SD); Rt, Reaction time; Tt, Total time*.

## Discussion

In post concussed athletes, compared to control participants, we found a lower mid-lateral open-eyed oscillation, significantly lower FetO_2_ average during the hypercapnic phase and significantly lower cerebral Δ[HbO_2_] during rest and recovery from a CPET test.

### Posture

The change in MLroo (eyes open) was chosen for the assessment of residual postural disorders as it could be referred to an altered modulation by the autonomic nervous system (Mirow et al., 2016) and be indicative of postural abnormalities in concussed participants. Surprisingly, in mTBI a significantly lower MLroo eo(eyes open) was observed in the sway test highlighting a higher postural efficiency in the mTBI group than in the control group, expressed by a decrease in the lateral displacement valued. In agreement with previous published experimental work (Fahr et al., 2015), this result can be explained by the higher level of training of these participants. In fact, considering the MLroo at closed eyes sway values, the difference between groups disappeared. So, it is therefore possible that the observed difference may be linked to the ability of professional athletes to maintain balance and decrease the medio-lateral oscillations thanks to long-lasting training (Fahr et al., 2015). Considering these results, the values of MLroo eo may not be adequate to make assessments of the effects of medium-long-term concussions in professional mTBI when compared to non-professional athletes or sedentary controls.

### Brain Oxygenation

Figure 1 reports the change of Δ[HbO_2_] and Δ[HHb] during the resting phase after a maximal effort during CPET in both groups. mTBI showed a noticeable drop in Δ[HbO_2_] compared to NC (*p* = 0.008) with much lower Δ hemoglobin at rest as well as reduced initial Δ oxygenation values compared to NC. Therefore, mTBI showed lower basal levels of oxygenated hemoglobin concentration, both at the beginning and at the end of the ramped test, with a constant decrease, and stabilization between the fourth and 5th min after. One of the possible causes of the physiological decline in both groups could be the redistribution of blood that takes place immediately after the change of the body posture, from supine to the sitting position leading to stabilization of the levels below the starting point when an orthostatic position is reached. However, this would not explain the lower Δ[HbO_2_] values of the mTBI group compared to NC (Ivan et al., 2004). No difference in Δ[HbO_2_] and Δ[HHb] between groups was observed during the hypercapnic test. Interestingly, in both groups of participants, Δ[HbO_2_] reached its final value after 5 min of the test, with a similar trend in NC and mTBI. However, the initial response to the stimulus was different in the two groups: in NC, which started from higher oxygenation values, a reduced increase in oxygen demand in the transcranial zone at the initial stage was observed starting from the 2nd min. In mTBI, which started from lower oxygenation levels, the oxygenation level gradually increased. In these participants a rather fast drop-down of the oxygenation occurred from the 4th min onwards, leading to Δ[HbO_2_] levels similar to those observed in NC. This is the consequences of the cerebral vasculature vasodilation (due to increased PaCO_2_). The observed differences may also be linked to different respiratory patterns observed in well-trained mTBI as they performed deeper respiratory acts with longer inspirations compared to controls.

As regards the changes of Δ[HbO_2_] and Δ[HHb] during the recovery phase after the cycle ergometer maximal test, a higher significantly different Δ[HbO_2_] decrease was observed in mTBI compared to NC. This observation could be attributed to a sub-clinical condition that leads to reduced cerebral perfusion in the time following efforts in concussed participants. These results agree with what observed by Bhambhani et al. in healthy subjects (Bhambhani et al., 2007) who experienced a collapse in cerebral oxygenation (Cox) during the final stage of maximal physical exercise to exhaustion. Another study that examines the prefrontal cortex oxygenation in healthy subjects belonging to another category of subjects is that of Rupp and Perrey (2008).

### Breathing Air-Flow

Recent studies have shown that cerebral oxygen extraction fraction (OEF) is an important physiological index also defined as an “index of the homeostasis of oxygen demand and supply in the human brain.” OEF has been found to be inversely related to FetCO_2_ (Jiang et al., 2019) in physiological conditions and is considered a potential clinical biomarker in neurodegenerative diseases such as Alzheimer's disease, multiple sclerosis and others (Jiang et al., 2019). As shown by some authors (Thomas et al., 2017), a decrease in OEF values has been reported in patients with mild cognitive impairment.

In our experimental conditions, we found a statistical difference in FetO_2_ between groups when the participants breathed spontaneously. The values of FetO_2_ and FetCO_2_, change from one breath to another in relation to the natural changes of the VT (Volume Tidal). For example, increasing VT will lead to an increase in FetO_2_ and a decrease in FetCO_2_ (Slessarev et al., 2007). In consequence, another reason that may have been influenced the variations in FetO_2_ is that it may have been induced by different respiratory characteristics and techniques observed in mTBI participants.

## Conclusion

Thanks to this study we can say that the medio-lateral variations evaluated using the MLroo parameter cannot sometimes be considered as a marker for long-term changes. If, as in our case, the participants who have suffered concussion turn out to be particularly trained, their balance values and therefore MLroo will also be particularly improved compared to a non-professional participant.

Similarly, the hypercapnia phase should not be the only phase considered about brain oxygenation, but from the results obtained, we can say that the rest and recovery phases are both of particular statistical interest showing a lower level of brain oxygenation. For this reason, it would be interesting to assess if these participants, trained with a specific physical activity, can modify their brain oxygenation levels.

Finally, the values of FetO_2_ should be considered in relation to the breathing method adopted by the participants, so it may be risky to rely only on this parameter.

### Limitations

There are limitations of the present work. One of the aspects that may have negatively affected the quality of the data, is related to the great variability between the participants, in particular regarding age. To ensure the high level of performance of these participants (national, European and World champions) we neglected the uniformity of personal data, inserting participants that had ± 9 years of difference between them. In addition to this, we had to group people from very different sport categories, thus having a difference of up to ± 15.62 kg in body mass.

Another limitation regards the equipment used for measuring the amount of Δ[HbO_2_] and Δ[HHb] that allowed to obtain measurements from the transcranial zone unlike the whole brain.

A limit of our study is we could not perform a confirming MRI imaging study for brain injury, because some participants refused to undergo it. This behavior was probably associated with the stigma and fear to have a brain injury and the discomfort of MRI.

Another limitation concerns the samples size that are limited because we tested available elitè athletes: the reason is that in Italy is not easy to find elitè athletes in this field of application; in addition, these athletes have to be concussed and this factor contributes to further reduce the sample.

Since we are investigating brain oxygenation, we considered male and female as comparable subjects, falling into the same group; we didn't consider sex as a discerning variable.

## Data Availability Statement

The raw data supporting the conclusions of this article will be made available by the authors, without undue reservation.

## Ethics Statement

The studies involving human participants were reviewed and approved by Ethics Committee of the University of Bologna, Italy. The patients/participants provided their written informed consent to participate in this study.

## Author Contributions

PT and AC conceived of the presented idea, developed the theory and performed the computations, verified the analytical methods, and carried out the experiment. AC encouraged PT to investigate mTBI and NIRS exams and supervised the findings of this work. PT wrote the manuscript with support from AC and GD'A. GD'A helped supervise the final version of the manuscript. All authors discussed the results and contributed to the final manuscript.

## Conflict of Interest

The authors declare that the research was conducted in the absence of any commercial or financial relationships that could be construed as a potential conflict of interest.

## Publisher's Note

All claims expressed in this article are solely those of the authors and do not necessarily represent those of their affiliated organizations, or those of the publisher, the editors and the reviewers. Any product that may be evaluated in this article, or claim that may be made by its manufacturer, is not guaranteed or endorsed by the publisher.

## Abbreviations

TBIs: Traumatic Brain Injuries
mTBIs: Mild TBIs
SRC: Sport Related Concussion
LOC: Loss Of Consciousness
AOC: Alteration Of Consciousness
PTA: Post-traumatic amnesia
PCS: Post-Concussion Syndrome
CTE: Chronic traumatic encephalopathy
PPCS: Persistent post-concussive symptoms
MCI: Mild Cognitive Impairment
HIC: Head Injury Criteria
GSI: Gadd Severity Index
SCAT: Sport Concussion Assessment Tool
Δ[HbO_2_]: Oxyhaemoglobin
Δ[HHb]: Deoxyhaemoglobin
tHb: Total hemoglobin
Cox: Cerebral Oxygenation
MLroo: Medio-Lateral oscillation
AProo: Anterior-Posterior range of oscillation
FetO_2_: Fraction End Tidal O_2_
OEF: Cerebral oxygen extraction fraction
MMA: Mixed Martial Arts
CBF: Cerebral Blood Flow.
